# Supplementary material for: Pediatric cerebrospinal fluid immune profiling distinguishes pediatric-onset multiple sclerosis from other pediatric-onset acute neurological disorders
Source: bioRxiv. 2025 May 23:2025.02.27.637541. Originally published 2025 Mar 1. Preprint. [Version 2] doi: 10.1101/2025.02.27.637541 (PMC11888486; doi:10.1101/2025.02.27.637541)
Supplement: Supplement 1 [file media-1.pdf]

SUPPLEMENTARY MATERIALS: SUPPLEMENTARY FIGURES AND LEGENDS & SUPLEMENTARY TABLES

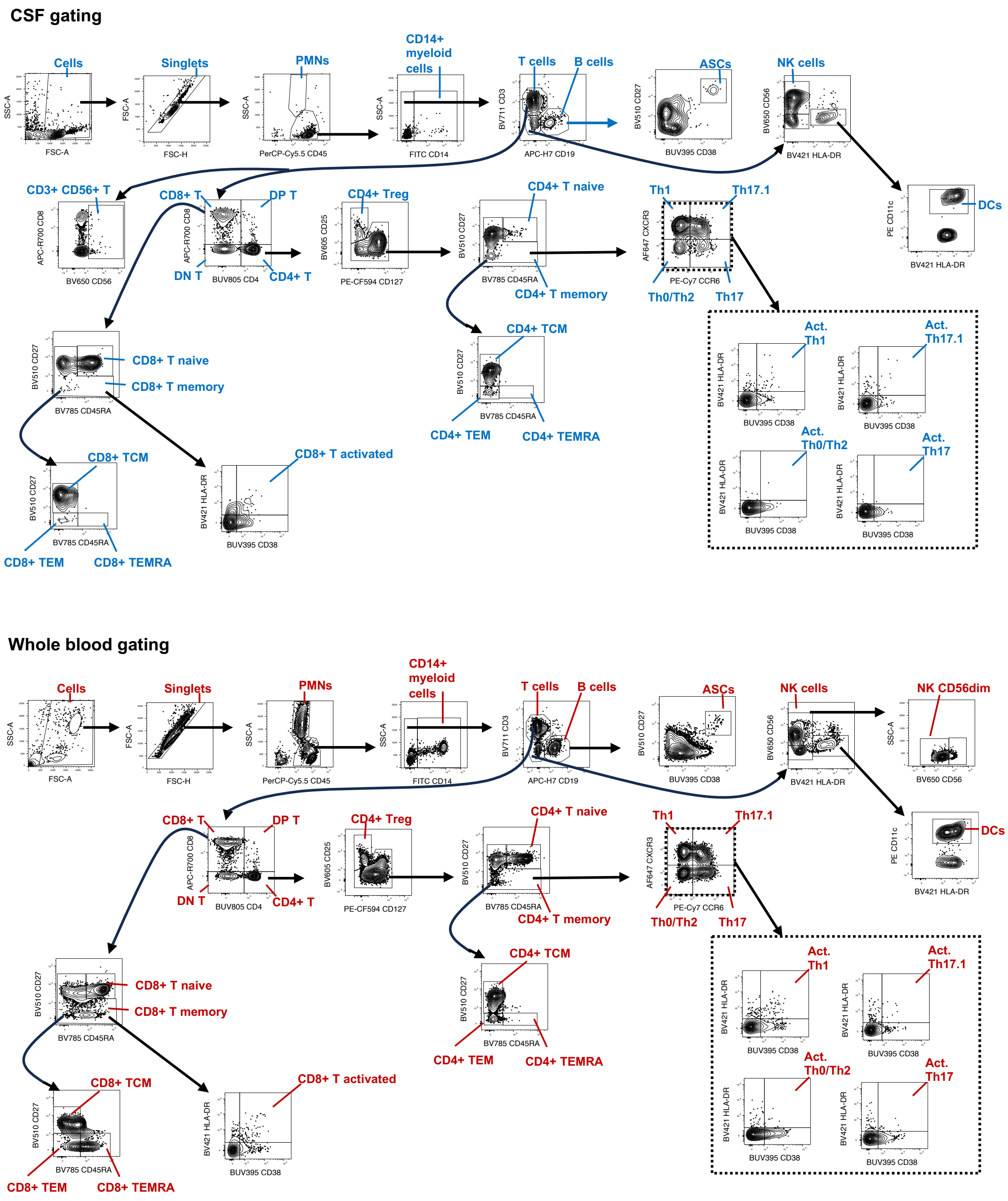

Supplementary Figure 1: Gating strategy for 16-color flow cytometric platform. Biaxial plots and gating strategy of flow cytometry results obtained using the 16-color flow cytometric platform for CSF and whole blood profiling in a representative example. Act. = activated.

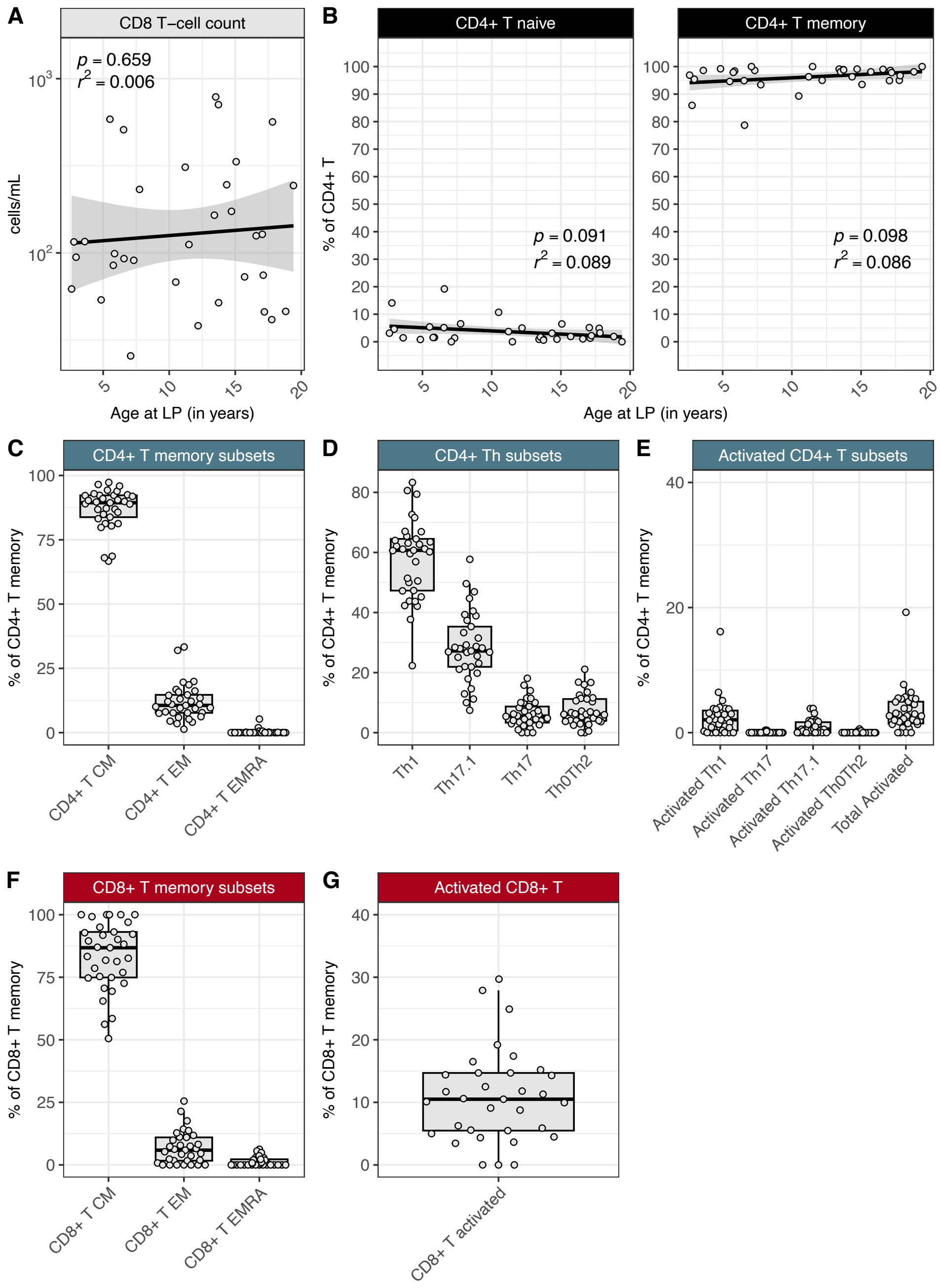

Supplementary Figure 2: T-cell subsets in NIND CSF. (**A**) Counts of total CD8+ T cells across the pediatric age-span in NIND CSF (n=33) with overlaid linear model. (**B**) Frequencies of naive and memory CD4+ T cells across the pediatric age-span in NIND CSF (n=33) with overlaid linear models. (**C**) Frequencies of central memory (CM), effector memory (EM), and effector memory CD45RA+ (EMRA) cells, as percent of memory CD4+ T-cells, in NIND CSF. (**D**) Distribution of

Th-status frequencies (based on CXCR3 and CCR6 expression) and their levels of activation based on HLA-DR/CD38 expression (**E**), as percent of memory CD4+ T-cells, in NIND CSF. (**F**) Frequencies of CM, EM, and EMRA cells as percent of memory CD8+ T-cells, in NIND CSF (n=33). (**G**) Frequencies of activated memory CD8+ T-cells in NIND CSF. NIND = non-inflammatory neurological disease.

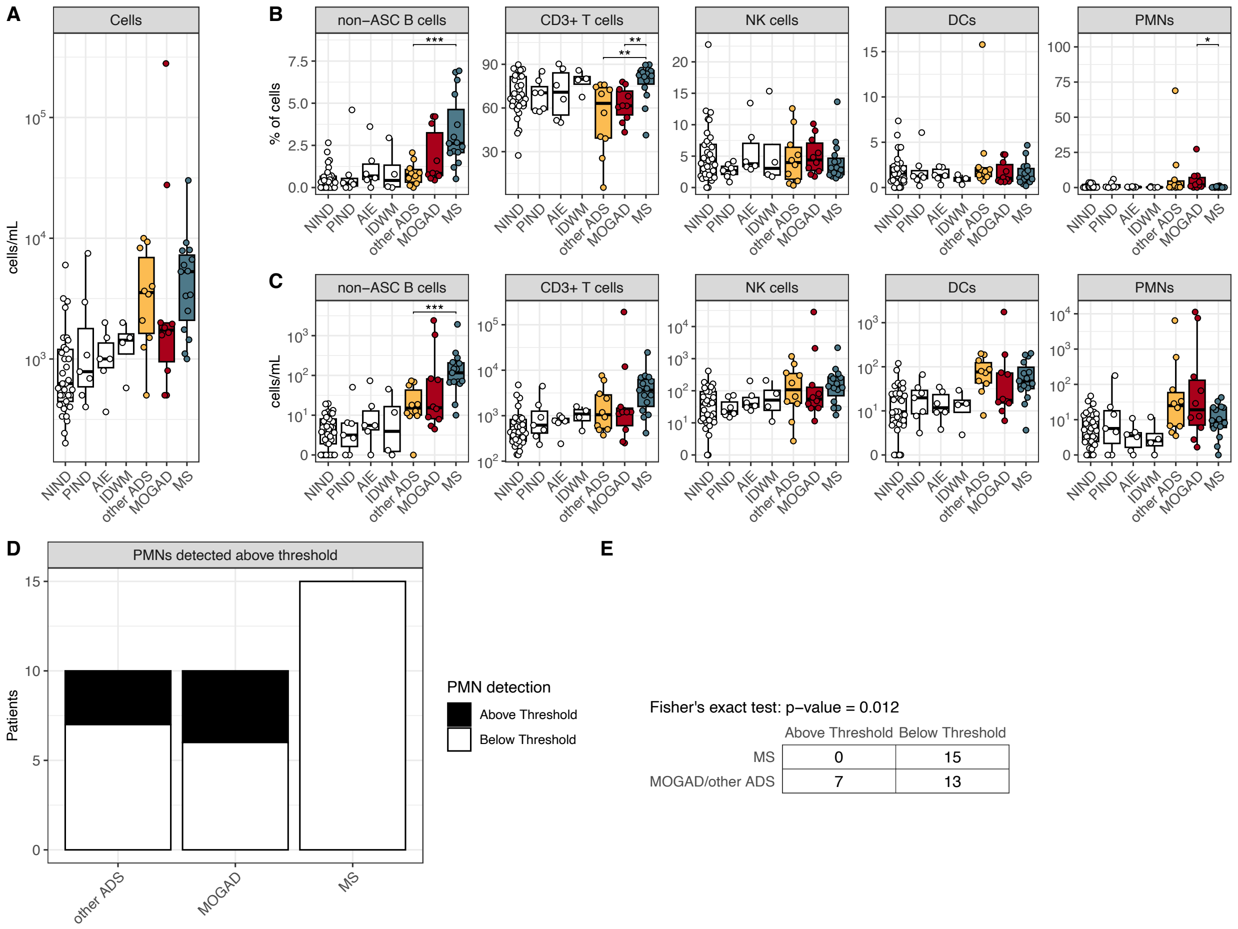

Supplementary Figure 3: CSF cell counts and population frequencies across NIND, PIND, AIE, IDWM, other ADS, MOGAD, and MS. (**A**) Cell counts (cells/mL) in NIND CSF (n=33), PIND CSF (n=7), AIE CSF (n=6), IDWM CSF (n=4), other ADS (n=10), MOGAD (n=10), and MS CSF (n=15). Frequencies (% of cells, **B**) and counts (cells/mL, **C**) of non-ASC B cells, CD3+ T cells, NK cells, DCs, PMNs across NIND, PIND, AIE, IDWM, other ADS, MOGAD, and MS CSF. (**D**) Frequency of PMN detection in patients above pre-defined threshold (see **Methods**) in other ADS, MOGAD, and MS CSF. (**E**) Fisher's exact test for the contingency table of PMN detection above pre-defined threshold, comparing MS CSF to MOGAD/other ADS CSF. For **Supplementary Fig. 3B-C**, Wilcoxon-rank-sum test used to compare MS to other ADS and MOGAD independently, and other ADS to MOGAD (* = p < 0.05, ** = p < 0.01, *** < 0.001,

**** < 0.0001). ASCs = antibody secreting cells., PMNs = polymorphonuclear cells NIND = non-inflammatory neurological disease, PIND = peripheral inflammatory neurological disease, AIE = autoimmune encephalitidies, IDWM = inherited disorders of white matter, other ADS = non-MS/non-MOGAD acquired demyelinating syndromes, MOGAD = myelin oligodendrocyte glycoprotein antibody-associated disease, MS = multiple sclerosis.

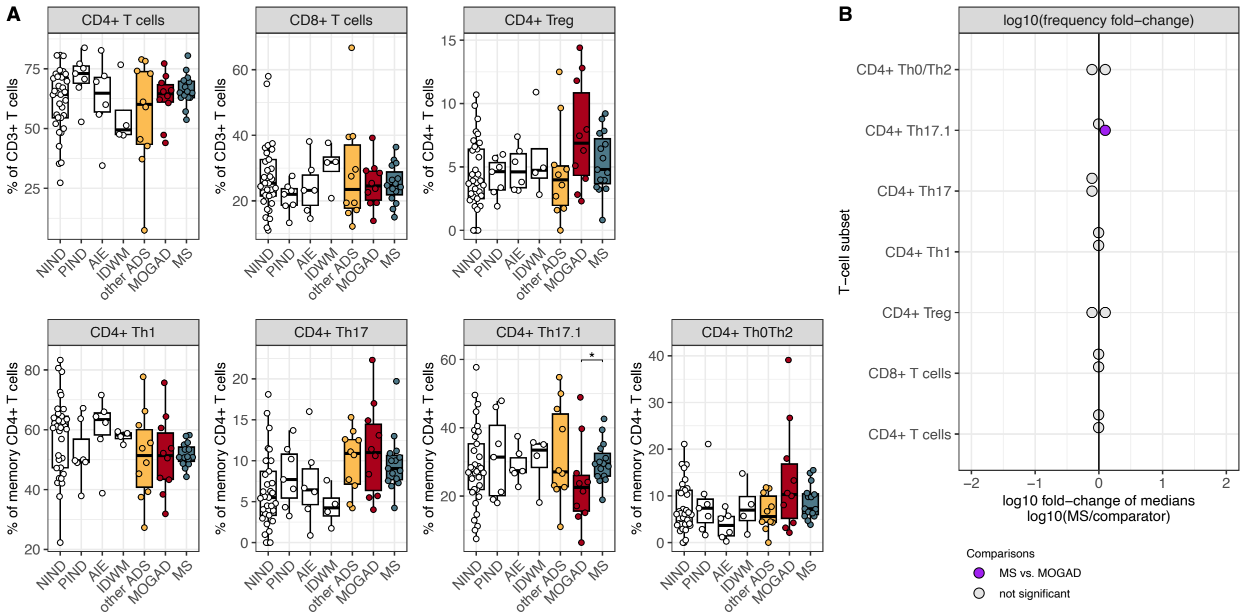

Supplementary Figure 4: CSF T-cell subset frequencies across NIND, PIND, AIE, IDWM, other ADS, MOGAD, and MS. (**A**) Frequencies of CD4+ T cells, CD8+ T cells, regulatory CD4+ T cells (CD4+ Treg), and CD4+ Th cell subsets in NIND CSF (n=33), PIND CSF (n=7), AIE CSF (n=6), IDWM CSF (n=4), other ADS (n=10), MOGAD (n=10), and MS CSF (n=15). (**B**) log10 of the fold change of median frequencies, comparing the median frequency in MS to the median frequency in other ADS and MOGAD for each T-cell subset. For **Supplementary Fig. 4A**, Wilcoxon-rank-sum test used to compare MS to other ADS and MOGAD independently, and other ADS to MOGAD (* = $p < 0.05$, ** = $p < 0.01$, *** < 0.001, **** < 0.0001). NIND = non-inflammatory neurological disease, PIND = peripheral inflammatory neurological disease, AIE = autoimmune encephalitidies, IDWM = inherited disorders of white matter, other ADS = non-MS/non-MOGAD acquired demyelinating syndromes, MOGAD = myelin oligodendrocyte glycoprotein antibody-associated disease, MS = multiple sclerosis.

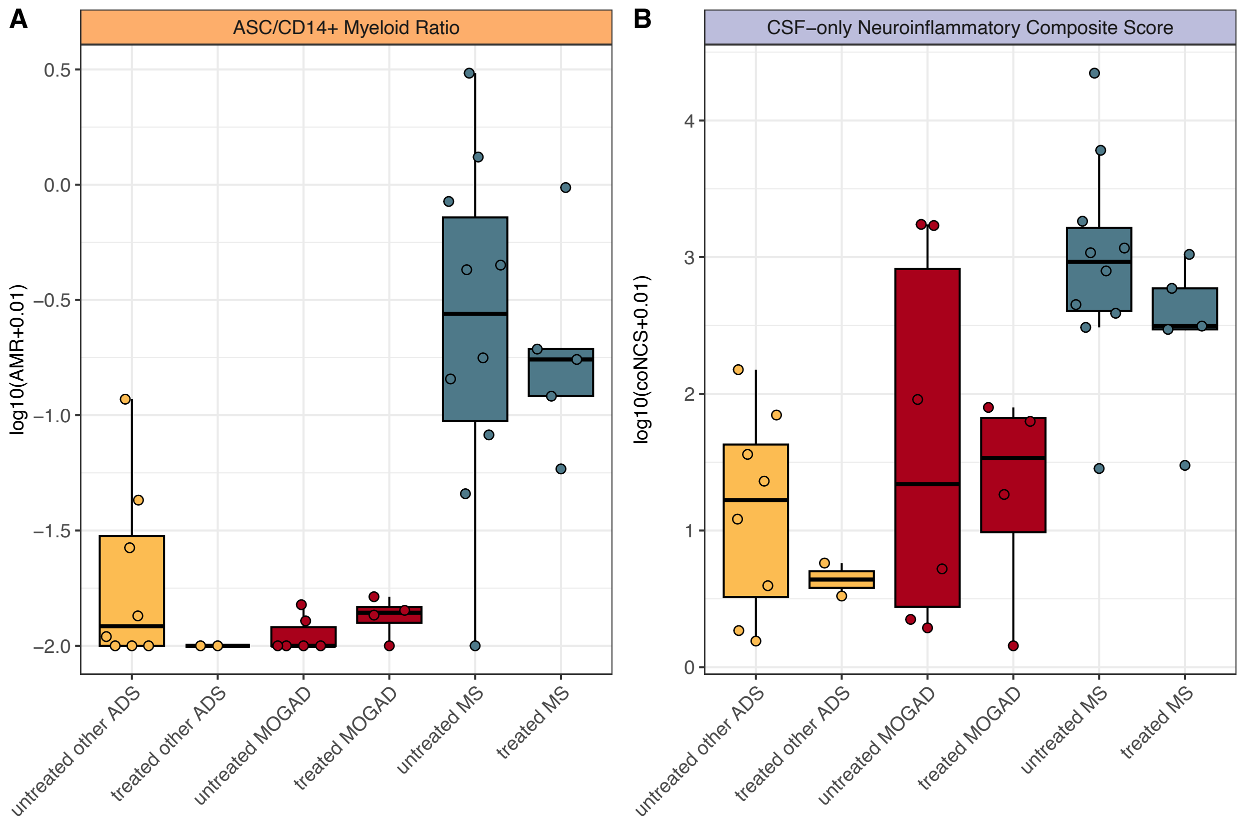

Supplementary Figure 5: AMR and coNCS stratified by treatment status within ADS diagnoses. (**A**) AMR and (**B**) coNCS across other ADS CSF (untreated n=8, treated n = 2), MOGAD CSF (untreated n=6, treated n=4), and MS CSF (untreated n=10, treated n=5). AMR = ASC to CD14+ myeloid cell ratio, coNCS = CSF-only neuroinflammatory composite score, other ADS = non-MS/non-MOGAD acquired demyelinating syndromes, MOGAD = myelin oligodendrocyte glycoprotein antibody-associated disease, MS = multiple sclerosis. “treated” indicates a CSF sample was drawn from a patient who had received any systemic glucocorticoid and/or IVIG treatment within 30 days prior to LP.

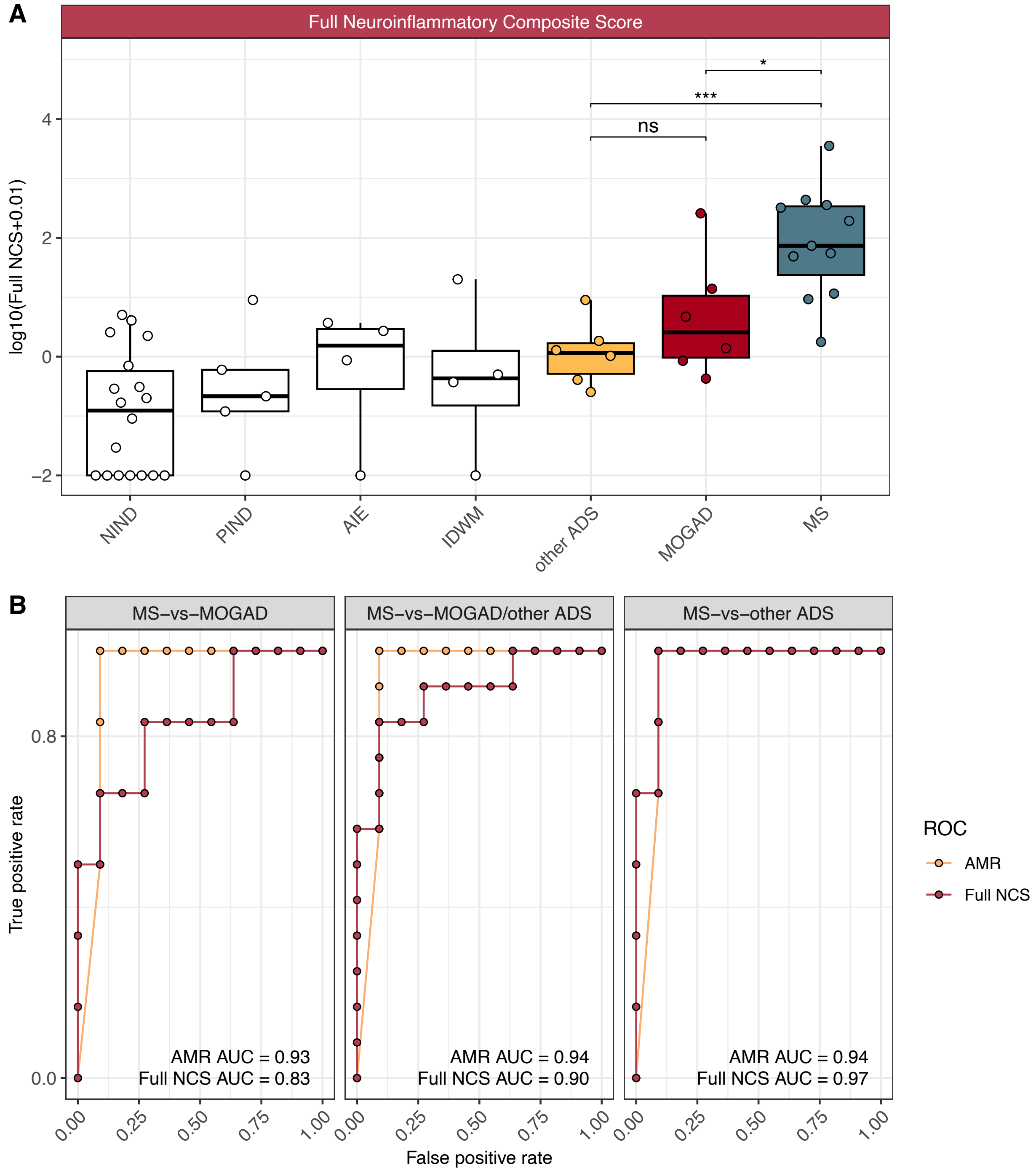

Supplementary Figure 6: Evaluation of AMR compared to full NCS classifier. (**A**) Values of the full NCS (utilizing blood measures, see **Methods**) across NIND CSF (n=18), PIND CSF (n=5), AIE CSF (n=4), IDWM CSF (n=4), and other ADS CSF (n=6), MOGAD CSF (n=6), and MS CSF (n=11). (**B**) Receiver operating characteristic (ROC) curves built for AMR or Full NCS classifiers

for the MS-vs-MOGAD/other ADS comparison, MS-vs-MOGAD comparison, and MS-vs-other ADS comparison, along with each corresponding area under the curve (AUC). For **Supplementary Fig. 6A**, Wilcoxon-rank-sum used to compare MS to MOGAD, MS to other ADS, and MOGAD to other ADS (* = $p < 0.05$, ** = $p < 0.01$, *** = $p < 0.001$, **** = $p < 0.0001$). AMR = ASC to CD14+ myeloid cell ratio, NCS = neuroinflammatory composite score, NIND = non-inflammatory neurological disease, PIND = peripheral inflammatory neurological disease, AIE = autoimmune encephalitidies, IDWM = inherited disorders of white matter, other ADS = non-MS/non-MOGAD acquired demyelinating syndromes, MOGAD = myelin oligodendrocyte glycoprotein antibody-associated disease, MS = multiple sclerosis.

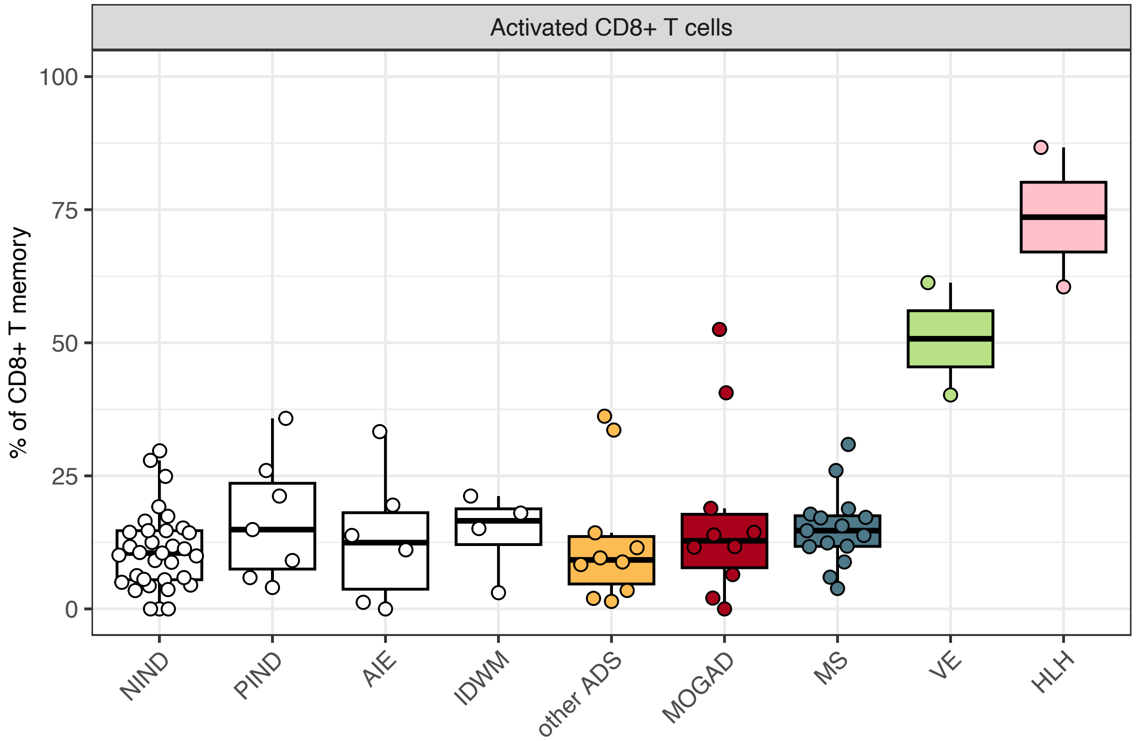

Supplementary Figure 7: Elevated frequencies of CSF CD8+ T-cell activation in viral encephalitis and hemophagocytic lymphohistiocytosis. CD8+ T-cell activation frequencies (as % of memory CD8+ T cells) in NIND CSF (n=33), PIND CSF (n=7), AIE CSF (n=6), IDWM CSF (n=4), other ADS (n=10), MOGAD (n=10), MS CSF (n=15), VE CSF (n=2), and HLH CSF (n=2). NIND = non-inflammatory neurological disease, PIND = peripheral inflammatory neurological disease, AIE = autoimmune encephalitidies, IDWM = inherited disorders of white matter, other ADS = non-MS/non-MOGAD acquired demyelinating syndromes, MOGAD = myelin oligodendrocyte glycoprotein antibody-associated disease, MS = multiple sclerosis, VE = viral encephalitis, HLH = hemophagocytic lymphohistiocytosis.

TABLE LEGENDS

Supplementary Table 1. Patient diagnoses and classification.

NIND = non-inflammatory neurological disease, PIND = peripheral inflammatory neurological disease, AIE = autoimmune encephalitidies, IDWM = inherited disorders of white matter, IIH = idiopathic intracranial hypertension, AIDP = acute inflammatory demyelinating polyneuropathy, CIDP = chronic inflammatory demyelinating polyneuropathy, GBS = Guillain-Barre syndrome, NMDARE = NMDA receptor encephalitis, HSV = herpes simplex virus, HLH = hemophagocytic lymphohistiocytosis, other ADS = non-MS/non-MOGAD acquired demyelinating syndromes, MOGAD = myelin oligodendrocyte glycoprotein antibody-associated disease, MS = multiple sclerosis, VE = viral encephalitis, HLH = hemophagocytic lymphohistiocytosis, ADEM = acute disseminated encephalomyelitis, ON = optic neuritis, TM = transverse myelitis, NOS = not otherwise specified.

Supplementary Table 2. Wilcoxon rank-sum test results for B-cell and CD14+ myeloid cell comparisons between ADS groups and NIND. NIND = non-inflammatory neurological disease, other ADS = non-MS/non-MOGAD acquired demyelinating syndromes, MOGAD = myelin oligodendrocyte glycoprotein antibody-associated disease, MS = multiple sclerosis.

Supplementary Table 3. MS score calculated for pediatric samples. MS score was cacluated as in Gross CC et al. and defined as plasma cells + intrathecal IgG synthesis, where: “plasma cells” = 1 if plasma cells are detected in CSF sample, 0 if not; “intrathecal IgG synthesis” = 1 if IgG synthesis rate elevated , 0 if not. Patient is classified as MS if their MS score is 2. IgG synthesis rate available for 9/10 other ADS patients, 7/10 MOGAD patients, and 15/15 MS patients. ASC

presence in pediatric dataset used as equivalence to plasma cell presence in MS score. Other ADS = non-MS/non-MOGAD acquired demyelinating syndromes, MOGAD = myelin oligodendrocyte glycoprotein antibody-associated disease, MS = multiple sclerosis.

Supplementary Table 4. 16-color flow cytometric panel targets, clones, fluorophores, dilution, and catalog numbers.

TABLES

Supplementary Table 1.

Category	Diagnosis
NIND	Altered mental status=4; Developmental delay=2; Developmental regression=2; Elevated optic nerves=2; Focal seizure=1; Headache=8; IIH=2; Landau Kleffner=1; Neuropathy=1; Papilledema=3; Pseudopapilledema=1; Psychiatric symptoms=6
PIND	AIDP=1; Bell's palsy=1; Bilateral anterior uveitis=1; CIDP=1; CN VI palsy=1; GBS=1; Myasthenia gravis=1
AIE	Antibody negative autoimmune encephalitis=3; NMDARE=3
IDWM	Aicardi-Goutieres syndrome=1; HMBS-related leukoencephalopathy=1; Unknown genetic leukodystrophy=2
ADS	Other ADS=10; MOGAD=10, MS=15
VE	Eastern equine virus encephalitis=1; HSV encephalitis=1
HLH	Isolated CNS HLH=1; XLP-HLH (due to SH2D1A mutation)=1

Supplementary Table 2.

population_name	tested_value	group1	group2	p	p.label
B cells	cells/mL	NIND	other ADS	0.00005	****
B cells	cells/mL	NIND	MOGAD	0.00096	***
B cells	cells/mL	NIND	MS	0.00000	****
CD14+ myeloid cells	cells/mL	NIND	other ADS	0.00026	***
CD14+ myeloid cells	cells/mL	NIND	MOGAD	0.01300	*
CD14+ myeloid cells	cells/mL	NIND	MS	0.84300	ns
B cells	% of cells	NIND	other ADS	0.01900	*
B cells	% of cells	NIND	MOGAD	0.00200	**
B cells	% of cells	NIND	MS	0.00000	****
CD14+ myeloid cells	% of cells	NIND	other ADS	0.89900	ns
CD14+ myeloid cells	% of cells	NIND	MOGAD	0.96600	ns
CD14+ myeloid cells	% of cells	NIND	MS	0.00000	****

Supplementary Table 3

Diagnosis	Patients with positive MS score	Total patients with available MS score
other ADS	0	9
MOGAD	1	7
MS	7	15

Supplementary Table 4

LASER NAME & FILTER	PARAMETER	TARGET	CLONE	DILUTION	CATALOG NUMBER
530/30 Blue [B]	FITC	CD14	MΦP9	1:20	BD 347493
710/50 Blue [A]	PerCP-Cy5.5	CD45	HI30	1:50	BD 564106
670/14 Red [C]	AF-647	CXCR3	G025H7	1:50	BioLegend 353712
730/45 Red [B]	APC-R700	CD8	SK1	1:50	BD 565192
780/60 Red [A]	APC-H7	CD19	SJ25-C1	1:50	BD 560177
450/50 Violet [F]	BV421	HLA-DR	G46-6	1:100	BD 562804
525/50 Violet [E]	BV510	CD27	L128	1:50	BD 563092
610/20 Violet [D]	BV605	CD25	2A3	1:50	BD 562660
660/20 Violet [C]	BV650	CD56	HCD56	1:50	BioLegend 318344
710/50 Violet [B]	BV711	CD3	SK7	1:50	BioLegend 344838
780/60 Violet [A]	BV786	CD45RA	HI100	1:50	BD 563870
379/28 UV [B]	BUV395	CD38	HB7	1:50	BD 563811
820/60 UV [A]	BUV805	CD4	SK3	1:50	BD 612888
586/15 YG [E]	PE	CD11c	Bu15	1:50	BioLegend 337206
610/20 YG [D]	PE-CF594	CD127	HIL-7R-M21	1:50	BD 562397
780/60 YG [A]	PE-Cy7	CCR6	11A9	1:50	BD 560620

REFERENCES

Gross CC, Schulte-Mecklenbeck A, Madireddy L, et al. Classification of neurological diseases using multi-dimensional CSF analysis. *Brain*. 2021;144(9):2625-2634. doi:10.1093/brain/awab147
